# Metformin induces autophagy and G0/G1 phase cell cycle arrest in myeloma by targeting the AMPK/mTORC1 and mTORC2 pathways

**DOI:** 10.1186/s13046-018-0731-5

**Published:** 2018-03-20

**Authors:** Yan Wang, Wenbin Xu, Zixun Yan, Weili Zhao, Jianqing Mi, Junmin Li, Hua Yan

**Affiliations:** Department of hematology, Rui Jin Hospital affiliated to Shanghai Jiao-Tong University School of Medicine, Shanghai, No. 197 Rui-Jin Er Road, Shanghai, 200025 China

**Keywords:** Myeloma, Metformin, AMPK, mTOR, Autophagy, Cell cycle arrest

## Abstract

**Background:**

Metformin is a commonly used drug for the treatment of diabetes. Accumulating evidence suggests that it exerts anti-tumor effects in many cancers, including multiple myeloma (MM); however, the underlying molecular mechanisms have not been clearly elucidated.

**Methods:**

The anti-myeloma effects of metformin were evaluated using human MM cell lines (RPMI8226 and U266) in vitro and in vivo NOD-SCID murine xenograft MM model. Cell viability was assessed with CCK8 and cell proliferation was measured by EdU incorporation assay. Cell cycle distribution and apoptosis were examined by flow cytometry. Transmission electron microscopy was used to visualized autophagosomes. Activation of AMPK and inhibition of mTORC1/C2 pathways was assessed by Western blot analysis. RPMI8226 cells and U266 cell lines with AMPK knockdown were generated by transfection with small interfering RNA targeting the AMPK-α1 and α2 subunits using Lipofectamine 2000 reagent.

**Results:**

Metformin effectively inhibited the proliferation of MM cell lines, an effect that was associated with the induction of autophagy and G0/G1 cell cycle arrest, but not apoptosis. Metformin activated AMPK and repressed both mTORC1 and mTORC2 signaling pathways in myeloma cells as well as downstream molecular signaling pathways, such as p-4EBP1 and p-AKT. AMPK activation resulted in direct phosphorylation and activation of tuberous sclerosis complex 2 (TSC2), leading to inhibition of the mammalian target of rapamycin (mTOR). In addition, metformin inhibited myeloma cell growth in an AMPK-dependent manner. The xenograft mouse model further confirmed that metformin inhibited tumor growth by upregulation of AMPK and downregulation of mTOR.

**Conclusions:**

Metformin inhibits the proliferation of myeloma cells by inducing autophagy and cell-cycle arrest. Our results suggest that the molecular mechanism involves dual repression of mTORC1 and mTORC2 pathways via AMPK activation. Our study provides a theoretical basis for the development of novel strategies for the treatment of MM using metformin as an already approved and safe drug.

## Background

Multiple myeloma (MM) is a clonal plasma cell proliferative disease. The incidence of MM has increased significantly in recent years and has now become the second-highest incidence hematological disease. Before the year 2000, MM was treated with combined chemotherapy regimens, such as melphalan plus prednisone, and the median survival of patients with MM was only 2 to 3 years [1]. Novel agents such as bortezomib, thalidomide, lenalidomide and high-dose chemotherapy followed by autologous stem cell transplantation (ASCT), have greatly improved outcomes, and the median survival of patients with MM reported to have increased to 4.6 years by 2005 and reaching 6.1 years by 2010 [2]. Nevertheless, MM remains incurable, and further development of novel and effective therapeutic drugs is urgently needed.

Metformin is a widely used drug administered orally to treat hypoglycemia, in particular for the treatment of type 2 diabetes mellitus (T2DM). Epidemiologic studies have suggested that metformin reduces the risk of cancers, such as breast, prostate, colon and pancreatic cancer, in patients with DM [3–5]. Furthermore, preclinical studies have shown that metformin inhibits cancer cell growth in vitro and in vivo [6, 7]. Recently, one study confirmed that metformin treatment for at least 4 years reduced the risk of progression of monoclonal gammopathy of undetermined significance (MGUS) to MM in diabetes patients [8]. Wu et al. also confirmed the association of metformin with improved outcomes in myeloma patients with DM [9].

The mechanism by which metformin reduces tumor incidence and lowers cancer-associated mortality remain unclear. Most studies suggest that activation of adenosine monophosphate activated protein kinase (AMPK) or/and reduction of serum insulin levels are the main mechanisms underlying the anti-cancer activity of metformin [7, 8, 10–13]. AMPK is an important energy-sensing enzyme involved in the maintenance of cellular energy homeostasis. Activated AMPK directly phosphorylates and activates tuberous sclerosis complex 2 (TSC2), leading to inhibition of the mammalian target of rapamycin complex 1 (mTORC1) signaling pathways [11–14].

The mTOR pathway is essential for tumor cell growth, proliferation and survival. Two mTOR complexes exist; mTORC1 and mTORC2 [15]. The mTORC1 complex consists of mTOR, raptor and mLST8 and mediates phosphorylation of S6K and 4EBP1, which stimulate mRNA translation and ultimately cell growth and proliferation. The mTORC2 complex, which consists of mTOR, rictor, mSIN1 and mLST8, is required for the phosphorylation of AKT at the Ser 473 residue located in the hydrophobic motif site [16]. First generation mTOR inhibitors, such as rapamycin, inhibit mTORC1, but the feedback loop of mTORC1/S6K axis mediates upregulation of AKT, which attenuates the anti-proliferative effect of rapamycin. Previous studies have shown that, unlike rapamycin, metformin inhibits mTOR without activating AKT [17, 18]. Thus, we hypothesized that metformin not only prevents phosphorylation of mTORC1 complex components, but also inhibits phosphorylation of AKT, a mTORC2 substrate, which is beneficial in the treatment of cancer.

To date, few studies have investigated the effects of metformin on hematological malignancy, especially MM. A recent study suggested that metformin inhibits the growth of myeloma cells by lowering serum insulin levels to inhibit signaling via the IGF/IGF-IR/PI3K pathway [19]. However, no convincing explanation of the role of the AMPK/mTORC1 and mTORC2 pathway in the anti-myeloma effects of metformin has been reported. In the present study, we investigated the role of the mTORC1/C2 signaling pathway and AMPK activation the mechanism underlying the anti-myeloma using human MM cell lines in vitro and an in vivo xenograft mouse model.

## Methods

### Cell lines and cultures

Human MM cell lines (RPMI8226 and U266) were purchased from the Cell Center of Chinese Academy of Sciences (Shanghai, China). Cells were cultured in RPMI-1640 medium (Sigma-Aldrich, St. Louis, MO, USA) supplemented with 10% heat-inactivated fetal bovine serum (FBS) (Sigma-Aldrich, St. Louis, MO, USA) at 37 °C humidified atmosphere containing 95% air and 5% CO2. Experiments were performed using cells in the logarithmic phase of growth.

### Reagents

Metformin (Sigma-Aldrich, St. Louis, MO, USA) was dissolved in phosphate-buffered saline (PBS) as a stock solution of 1 M. Primary antibodies for specific detection of p-AMPK (Thr172), AMPK, Phospho-Tuberin/TSC2 (Ser1387), p-mTOR (Ser2481), p-mTOR (Ser2448), mTOR, p-p70S6K (Thr389), p70S6K, p-4EBP1 (Thr37/46), 4EBP1, p-AKT (Ser473), and AKT, as well as horseradish peroxidase (HRP)-conjugated anti-rabbit secondary detection antibodies, were purchased from Cell Signaling Technology (Beverly, MA, USA). Antibodies against cyclin D1, p21, p27 and the AMPK inhibitor compound C were all obtained from Abcam (Cambridge, UK). The autophagy inhibitor 3-methyladenine (3-MA) was obtained from Sigma-Aldrich (St. Louis, MO, USA).

### Cell viability and proliferation assays

Cell viability was assessed with Cell Counting Kit-8 (CCK-8, CK04–500, Dojindo, Kumamoto, Japan). Cells were seeded in 96-well plates (2 × 10^4^/well) and incubated without or with metformin at the indicated concentrations (0, 5, 10, 20, 40, and 80 mM) at 37 °C for 24, 48 and 72 h. Subsequently, cells were incubated for an additional 2 h with 10 μl of CCK-8 at 37 °C. Absorbance values were determined at a wave length of 450 nm by spectrophotometric measurements (Molecular Devices Corp., Sunnyvale, CA, USA). Cell proliferation was assessed using a Cell Light 5-ethynyl-2′-deoxyuridine (EdU) imaging kit (C103103, RiboBio, Guangzhou, China) according to the manufacturer’s instructions, and cells were examined under a fluorescence microscope.

### Cell cycle assay

Cells (RPMI8226 and U266) were seeded at 1×10^6^ cells per well in 6-well plates and incubated without or with metformin (5 mM, 20 mM) for 24 and 48 h. Cells were harvested and permeabilized overnight with pre-cooled 75% ethanol at 4 °C. Cells were then treated with 1 mg/ml RNase A for 30 min at 37 °C and stained with 50 μg/ml propidium iodide in the dark for 15 min. Cells were then analyzed by flow cytometry (FACSCalibur, BD Biosciences, Bedford, MA, USA).

### Cell apoptosis assay

Cell apoptosis was measured using an Annexin V-FITC/PE Apoptosis kit (BD Biosciences, San Jose, CA, USA) according to the manufacturer’s instructions. Briefly, cells were harvested and washed with PBS buffer and resuspended in 100 μl binding buffer. Annexin V-FITC (5 μl) was then added and the cell suspension was incubated in the dark for 5 min before incubation for a further 15 min in the dark in the presence of 5 μl propidium iodide. Fluorescence intensity was measured by flow cytometry (FACSCalibur, BD Biosciences).

### Small interfering RNA transfection

RPMI8226 and U266 cells were transfected with small interfering RNA (siRNA) targeting the AMPK-α1 and α2 subunits (sc-45,312; Santa Cruz, CA, USA) or scrambled siRNA (sc-37,007; Santa Cruz, CA, USA) as a control. Briefly, cells in each well were transfected with 30 pmol siRNA using the Lipofectamine 2000 Transfection Reagent (11,668; Invitrogen, Carlsbad, CA, USA) according to the manufacturer’s instructions. After transfection for 6 h, the culture medium was replaced with PRMI1640, and 20 mM metformin was subsequently added, followed by incubation for a further 24 h.

### Western blot analysis

Treated and untreated cells (RPMI8226 or U266) were harvested and lysed in 200μl lysis buffer (Cell Signaling, Beverly, MA, USA). After quantification, protein extracts were separated on 5%–15% sodium dodecyl sulfate-polyacrylamide gel electrophoresis (SDS-PAGE), transferred to a polyvinylidene difluoride (PVDF) membrane. Subsequently, membranes were blocked with 5% non-fat dried milk in Tris-buffered saline-Tween 20 (TBS-T, 20 mM Tris, PH 7.6, 137 mM NaCl, and 0.1% Tween 20) for 30 min at room temperature. The membranes were then washed and incubated with the appropriate primary antibody overnight at 4 °C. The next day, membranes were washed and incubated with horseradish peroxidase-conjugated secondary antibody in TBS-T at room temperature for 2 h. The immunocomplexes were visualized using Millipore’s enhanced chemiluminescence detection system (ChemiDoc Touch, BioRad).

### Transmission electron microscopy

MM cells (RPMI8226 and U266) were cultured in the presence of media or 20 mM metformin for 6, 12 and 24 h. Cells were harvested and fixed overnight at 4 °C in 4% glutaraldehyde and rinsed with 0.1 M cacodylate buffer. The cells were then fixed in 1% osmium tetroxide for 2 h at 4°C, dehydrated in a graded series of ethyl alcohol, and embedded in resin. Ultrathin sections were prepared, stained with uranyl acetate and lead citrate, and examined on a Philips CM120 transmission electron microscope (Eindhoven, The Netherlands).

### Xenograft tumor model

A murine xenograft myeloma model was applied to evaluate the anti-tumor efficiency of metformin in vivo. NOD/SCID mice (aged 5 weeks) were obtained from the Shanghai Laboratory Animal Center (Shanghai, China). Mice were injected subcutaneously into the right flank with 1 × 10^7^ RPMI8226 cells suspended in 100 μl PBS buffer. After approximately two weeks, when tumors reached a size of approximately 0.5 cm × 0.5 cm, 12 mice were randomly assigned into two cohorts (six mice per cohort). Control mice were administered PBS orally (control cohort), and the remaining received metformin orally every day (250 mg/kg/day). The mice were monitored for body weight and tumor volume on every three day. Tumor volume was calculated as 0.5 × (length) × (width)^2^. Mice were sacrificed by cervical dislocation after 21 days of treatment. Tumors were dissected and frozen in liquid nitrogen or fixed in formalin. All the procedures used in these experiments were approved by the Shanghai Jiao Tong University School of Medicine Institutional Animal Care and Use Committee.

### Immuno histochemistry

Immunohistochemical staining was performed on paraffin embedded sections (thickness, 5 μm) of the mouse xenograft tumors. The sections were stained using an indirect immunoperoxidase method with antibodies for specific detection of phosphor-AMPK (1:100), phosphor-mTOR (1: 100) (CST, Beverly, MA, USA)) and Ki-67 (1 : 500) (Dako, Glostrup, Denmark). Expression levels were scored semi-quantitatively based on the percentage of positive cells according to the following system: +, < 25%; ++, 25%–49%; +++, 50%–74%; ++++, 75%–100%.

### Statistical analysis

In vitro experiments were performed in triplicate and the results were presented as mean ± standard deviation (SD). Variations between the experimental groups were determined by Student’s t-test. *P* values < 0.05 were considered to statistical significance. Data was analyzed using GraphPad prism software (San Diego, CA, USA).

## Results

### Metformin inhibits cell proliferation in human myeloma cell lines

**Fig. 1:**
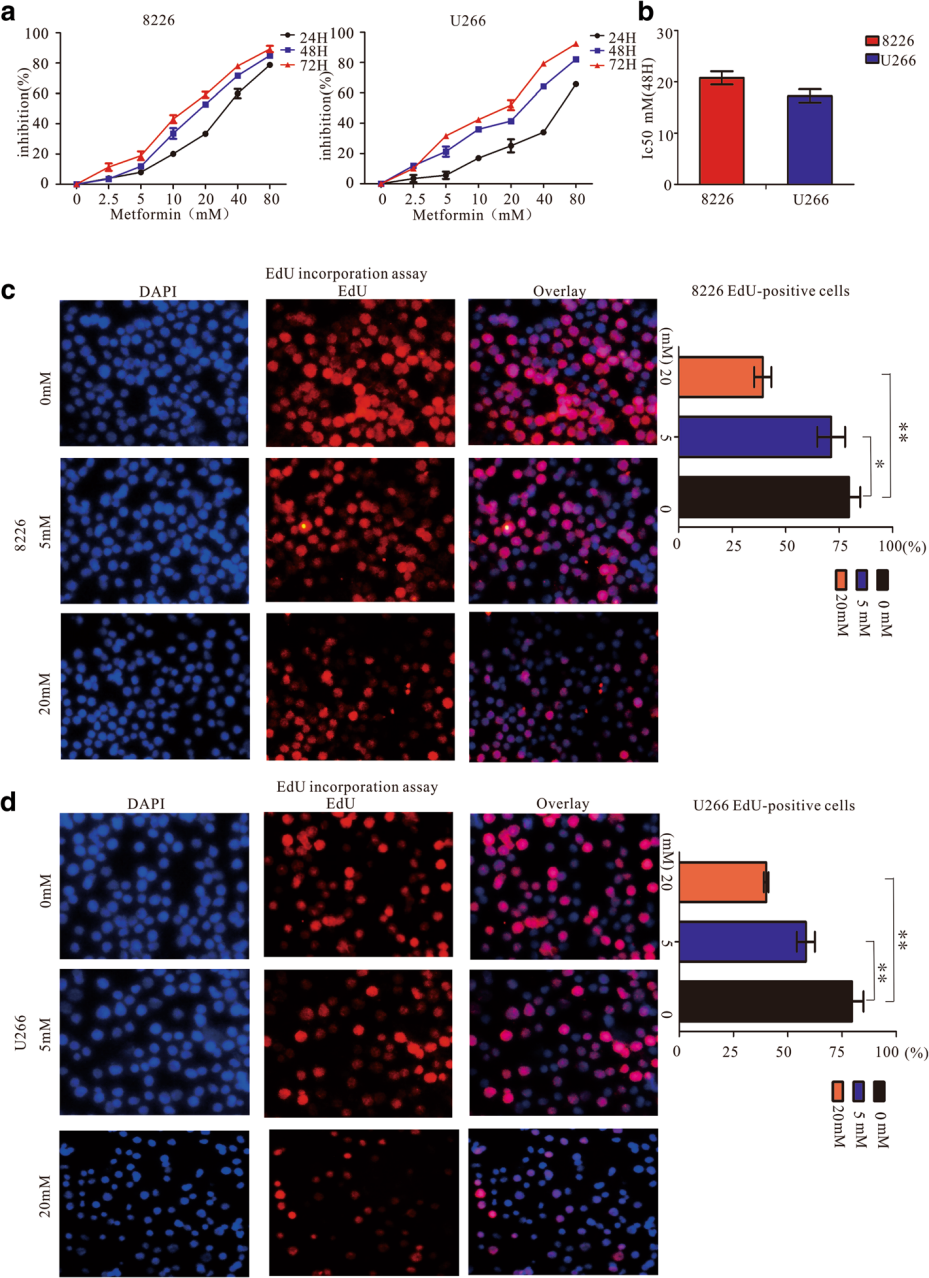
Metformin inhibits cell proliferation in human MM cells. **a** Cell viability was assessed by CCK8 assay. RPMI8226 and U266 cells were treated with 0, 2.5, 5, 10, 20, 40 or 80 mM metformin for 24, 48 and 72 h. **b** Fifty percent growth-inhibitory concentrations (IC50) assay results obtained in MM cell lines after treatment with metformin for 48 h. **c**, **d** Cell proliferation analysis by EdU incorporation assay. RPMI8226 and U266 cells were treated with 0, 5 mM, and 20 mM metformin for 24 h. The percentage of EdU positive cells. All data are expressed as the mean ± SD of values from triplicates experiments. **P < 0.01 and ***P < 0.001 compared with the control group

To investigate the effect of metformin on myeloma cell growths, RPMI8226 and U266 cells were treated with different concentrations of metformin for 24, 48 and 72 h. Cell viability was evaluated using a CCK-8 assay. As shown in Fig. 1a, cell viability decreased with increasing concentrations of metformin and with increasing duration of treatment. The 50 % growth-inhibitory concentrations (IC50) after treatment with metformin for 48 h was 20.2 ± 1.2 mM for RPMI8226 cells and 17.9 ± 1.1 mM for U266 cells (Fig. 1b). The effect of metformin on cell proliferation was further evaluated by 5-ethynyl-2′-deoxyuridine (EdU) incorporation assay. After treatment with 5 mM or 20 mM metformin for 24 h, EdU staining was performed for both cell lines. The percentage of EdU-stained cells was calculated on the basis of five randomly selected fields for each group. The percentage of cell proliferation decreased significantly with increasing concentrations of metformin (Fig. 1c-d). These results suggested that metformin inhibited the growth of human myeloma cell lines in vitro.

### Metformin induces G0/G1 phase cell cycle arrest, but did not induce apoptosis in myeloma cells

**Fig. 2:**
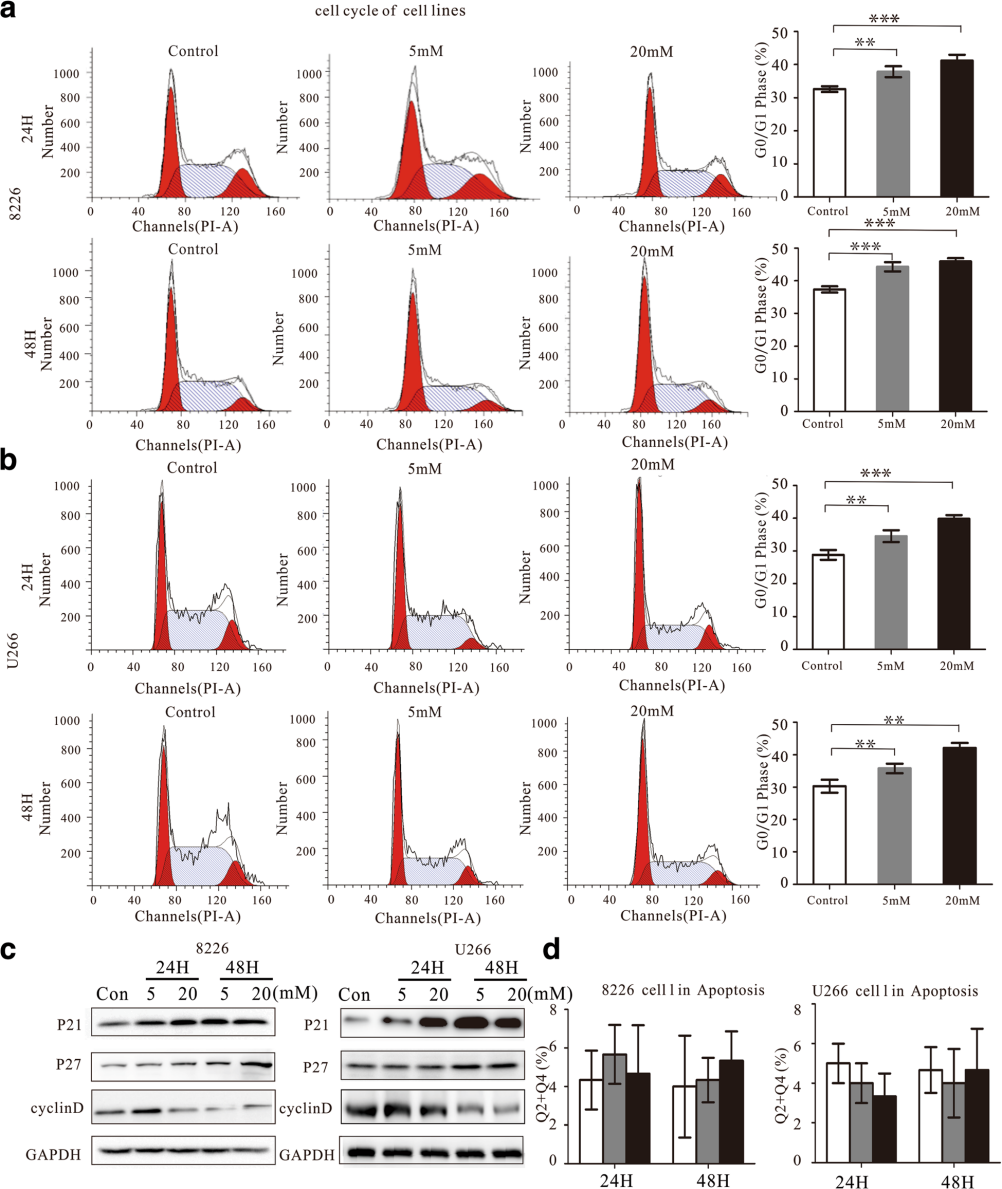
Metformin induces G0/G1 cell cycle arrest, but not apoptosis in myeloma cell lines. **a**, **b** Representative results showing the distribution of cells in G0/G1, S, or G2 phase in RPMI8226 and U266 cells following treatment or with metformin (5 mM or 20 mM) or without for 24 and 48 h. Histograms showing the percentage of myeloma cells in G0/G1, S, and G2 phases. **c** Western blot showing repression of the G1 phase-related cell cycle regulatory proteins cyclin D1 and activation of p21^CIP1^ and p27^KIP1^ . **d** Histograms showing the percentage of apoptotic RPMI8226 and U266 cells following treatment with metformin (0, 5, 20 mM) for 24 and 48 h, as detected by flow cytometry. All the experiments were performed in triplicated and repeated on at least three occasions. **P < 0.01 and ***P < 0.001 compared with the control group

To investigate how metformin influences myeloma cell growths, we analyzed cell cycle and apoptosis. RPMI8226 and U266 cells were treated with metformin (0 mM, 5 mM, and 20 mM) for 24 h. Flow cytometric analysis of propidium iodide (PI) stained cells revealed accumulation cells in the G0/G1 phase, while the fraction of cells in the S phase decreased (Fig. 2a-b). Western blot analysis of the levels of the main cell cycle regulatory proteins following metformin treatment of RPMI8226 and U266 cells clearly showed downregulation of cyclin D1, while p21^CIP1^ and p27^KIP1^ were upregulated (Fig. 2c). The pro-apoptotic effects of metformin were measured by flow cytometric analysis of annexin V-FITC/PE staining. As shown in Fig. 2d, metformin did not induce apoptosis of myeloma cells compared with the effects of the medium control. These results indicated that metformin inhibited the growth of RPMI8226 and U266 cells by blocking the cell cycle progression in the G0/G1 phase.

### Metformin inhibits the growth of myeloma cells partially by inducing autophagy

**Fig. 3:**
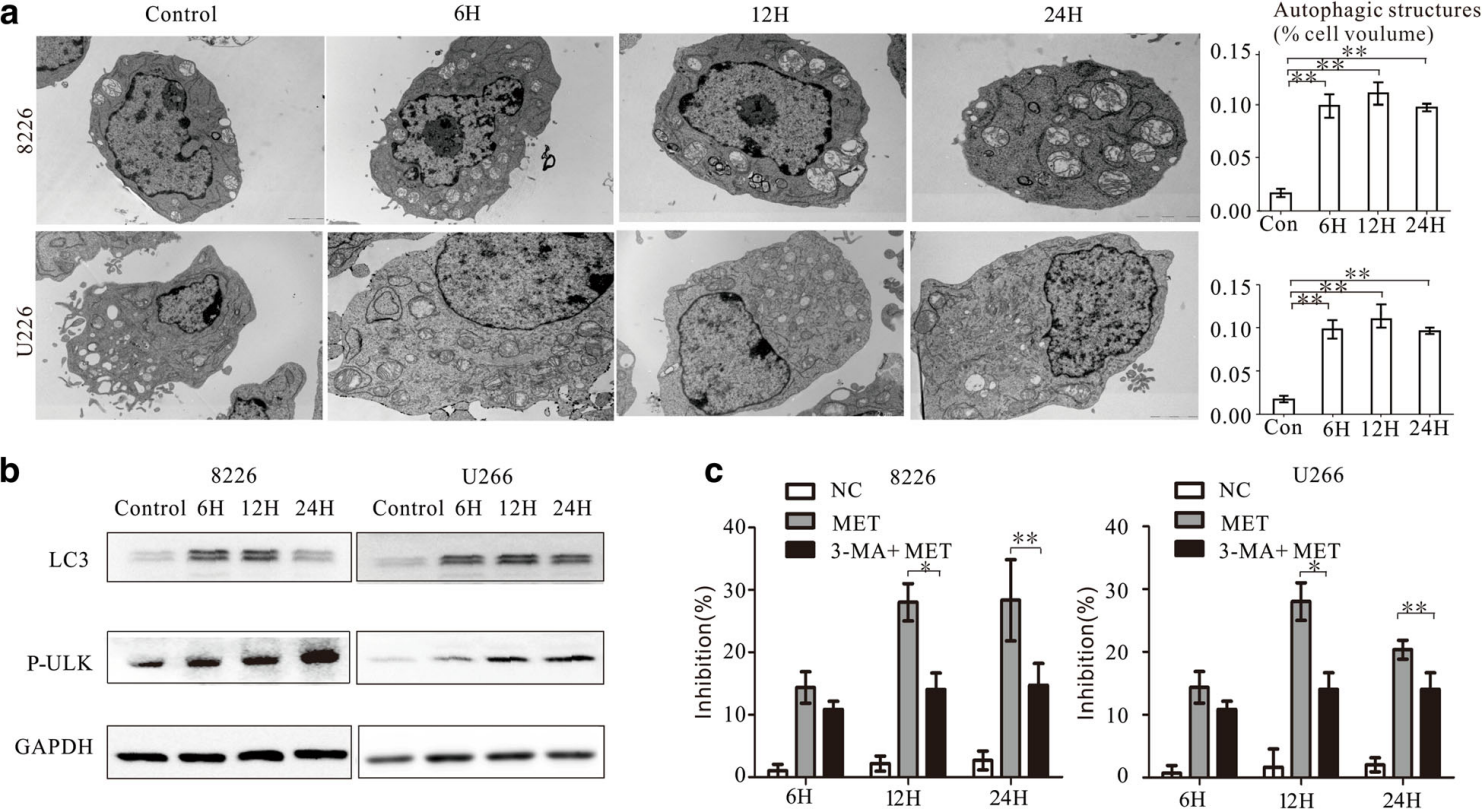
Autophagy enhancement contributes to the inhibitory effect of metformin in myeloma cells. **a** Transmission electron microscopy images showing the formation of autophagosomes in RPMI8226 and U266 cells after treatment with metformin 20 mM for 6, 12, and 24 h. Histograms show the percentage of autophagic structures in the total cell volume. **b** Western blot showing the effect of metformin treatment on expression of the autophagy-related protein LC3-II. **c** The inhibitory effect of metformin on myeloma cells was attenuated by pre-treatment with the autophagic inhibitor 3-MA. ** *P* < 0.01 and ***P < 0.001 compared with the control group

Previous studies have suggested that metformin induces autophagy. Transmission electron microscopy (TEM) showed the presence of numerous typical double- membraned autophagic vesicles (autophagosomes) in RPMI8226 and U266 cells following treatment with metformin (Fig. 3a). The expression of autophagy-related LC3-II was notably higher after treatment with metformin for 6, 12, and 24 h (Fig. 3b). To confirm the contribution of autophagy to the observed inhibition of growth, RPMI8226 and U266 cells were pre-treated with the autophagic inhibitor 3-methyladenine (3-MA) for 1 h prior to the addition of 20 mM metformin for 6, 12, and 24 h. The results showed that 3-MA partially rescued myeloma cells from the inhibitory effects of metformin, indication that the growth-inhibitory effect of metformin is associated with autophagy (Fig. 3c).

### Metformin activates AMPK and represses both the mTORC1 and mTORC2 signaling pathways in myeloma cells

**Fig. 4:**
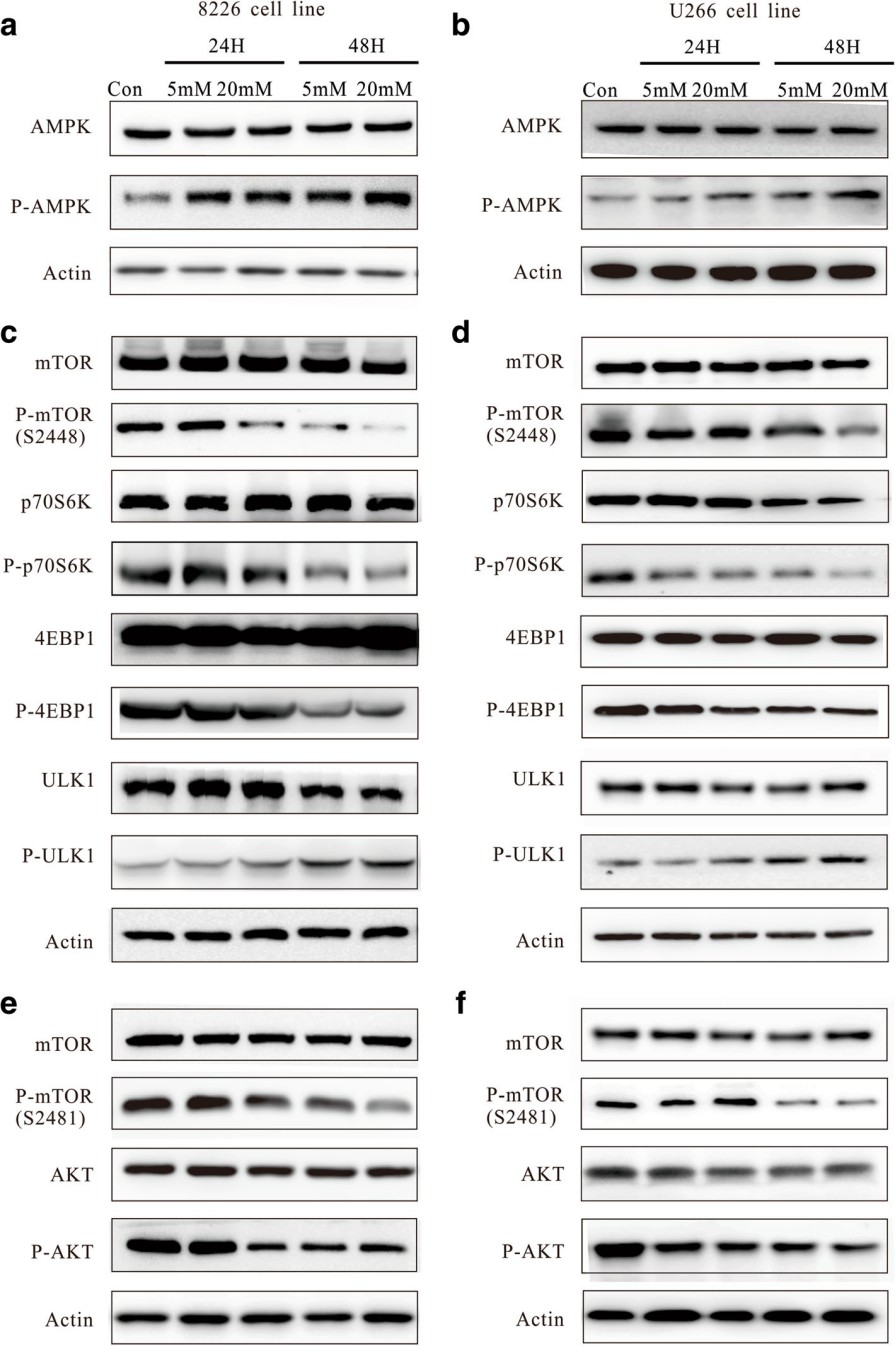
Metformin activates AMPK and represses both the mTORC1 and mTORC2 pathways in myeloma cells. RPMI8226 and U266 cells were treated without or with metformin (5 mM or 20 mM) for 24 and 48 h. **a**, **b** Western blot showing expression of AMPK and p-AMPK. **c**, **d** Western blot showing expression of mTORC1-mediated signaling pathway proteins. **e**, **f** Western blot analysis of expression of mTORC2-mediated signaling pathway proteins

Metformin has been reported to activate AMPK, which is considered to be a key molecular that controls cell growth, proliferation, and autophagy. As shown in Fig. 4a and b, we confirmed that phosphorylated AMPK (p-AMPK) expression was upregulated in RPMI8226 and U266 cells following treatment with metformin (5 mM, 20 mM) for 24, 48 h. Further investigation of the mTORC1 and mTORC2 signaling pathways showed that metformin downregulated phosphorylation of mTOR at Ser2448, leading to repression of mTORC1. This subsequently repressed the phosphorylation of key proteins involved in transcription, such as eukaryotic initiation factor 4E binding protein 1 (4EBP1) at Thr37/46, and p70S6 kinase 1 (p70S6K1) at Thr389. Inhibition of mTORC1 also upregulated expression of the autophagy-associated protein, unc-51-like kinase 1 (ULK1) (Fig. 4c and d). As shown in Fig. 4e and f, mTOR phosphorylation at Ser2481 (which is a marker of mTORC2 activation) was downregulated, leading to downstream repression of AKT phosphorylation at Ser473 and ultimately, to the inhibition of the myeloma cell growths.

### Metformin inhibits myeloma cell growth in an AMPK-dependent manner

**Fig. 5:**
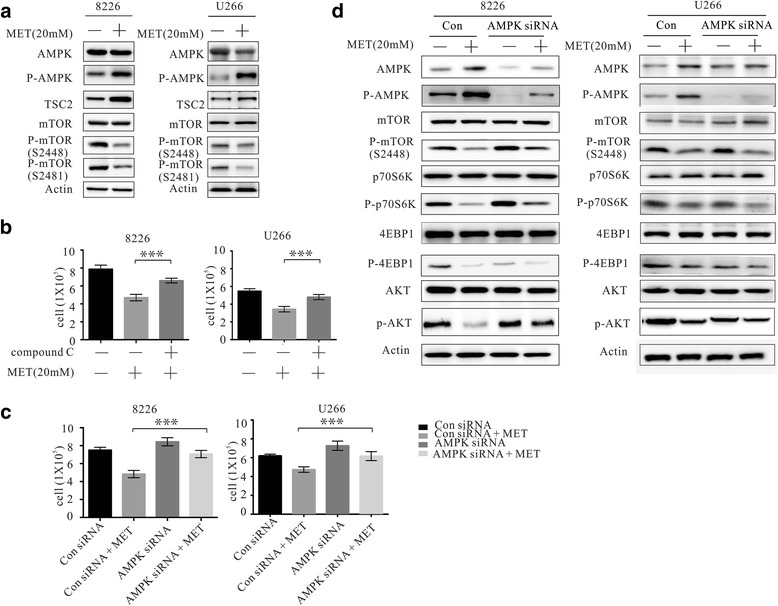
Metformin inhibits growth of myeloma cells in an AMPK-dependent manner. **a** Western blot showing that metformin activates AMPK and upregulated TSC2 to inhibit mTOR in RPMI8226 and U266 cells. **b** CCK8 assay showing that the specific AMPK inhibitor compound C abrogated the metformin-induced RPMI8226 and U266 cells growth inhibition. **c** CCK8 assay showing that siRNA-mediated knockdown of AMPK in RPMI8226 and U266 cells attenuated metformin-induced cell growth inhibition. **d** Western blot analysis showing that siRNA-mediated knockdown of AMPK in RPMI8226 and U266 cells failed to inhibit phosphorylation of the mTORC1/C2 signaling pathways. *P < 0.05 and **P < 0.05 compared with the control group

Previous studies suggested that metformin inhibits mTOR in either an AMPK-dependent or AMPK-independent manner. The potential association between repression of the mTORC1/C2 pathway in myeloma cells AMPK activation remains to be established. It has been reported that AMPK negatively regulates the mTOR signaling pathway by activating TSC2 through phosphorylation of Ser1387. In this study, we analyzed the levels of p-AMPK and its primary substrate, TSC2, by western blot. After treatment with 20 mM metformin for 24 h, the levels of both p-AMPK and p-TSC2 was upregulated, while both p-mTOR (Ser2481) and p-mTOR (Ser2448) levels were downregulated (Fig. 5a). To further confirm the role of AMPK activation in the mechanism responsible for the growth-inhibitory effect of metformin on myeloma cells, the specific AMPK inhibitor, compound C, and the AMPK-specific siRNA was used to block AMPK expression in RMPI8226 and U266 cells. After pre-treatment with compound C, metformin-induced inhibition of myeloma cell growth was attenuated (Fig. 5b). Similarly, the molecular knockdown of AMPK abrogated metformin-induced inhibition of myeloma cell growth and attenuated metformin-induced repression of mTORC1/C2 signal pathway (Fig. 5c and d). Thus, our results indicate that metformin inhibits myeloma cell growth by repressing mTORC1/C2 signaling pathway in an AMPK-dependent manner.

### Metformin shows inhibitory activity against myeloma in a NOD/SCID mouse xenograft model in vivo

**Fig. 6:**
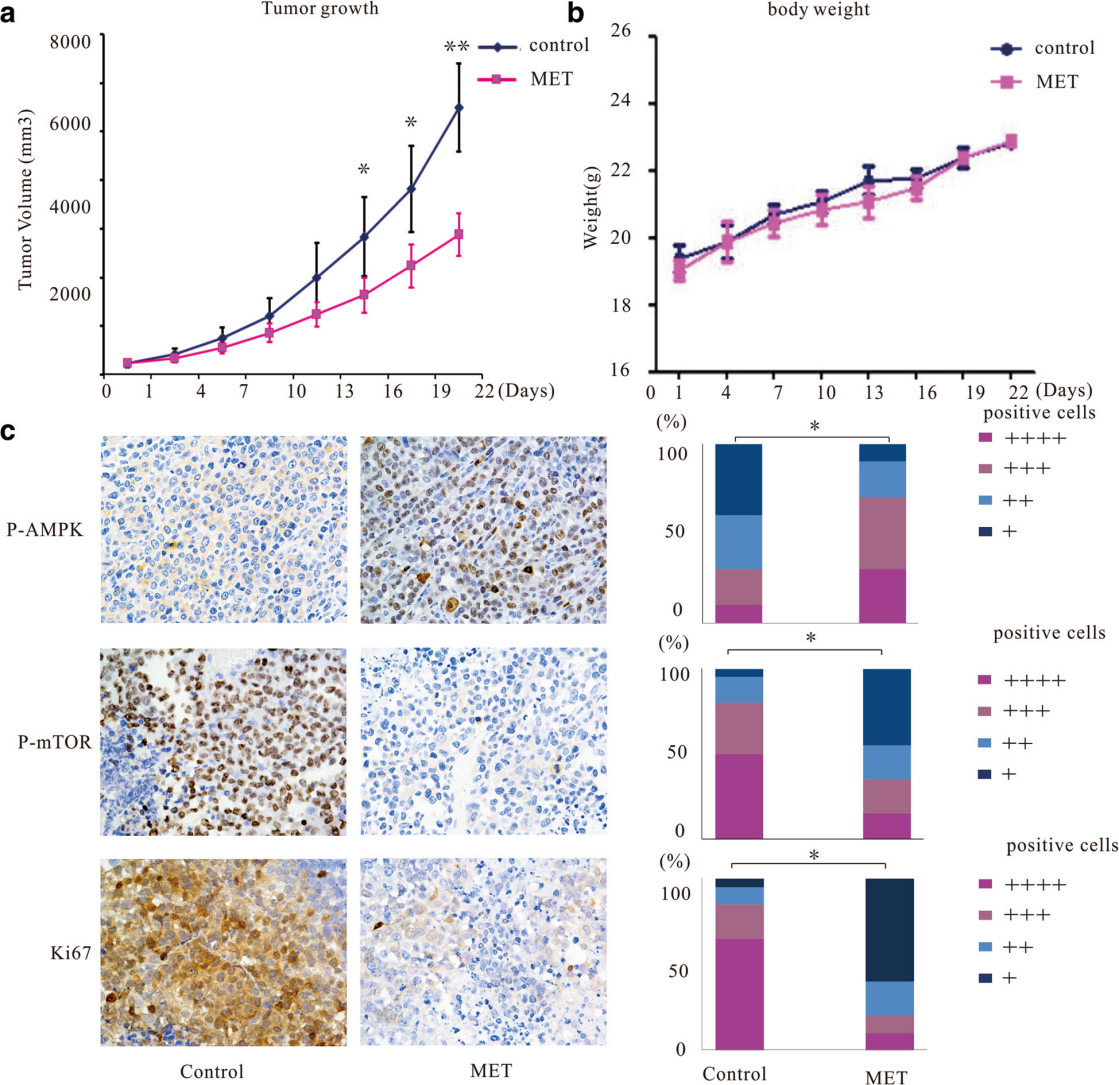
Metformin inhibits tumor growth in an RPMI8266 xenograft mouse model. **a** NOD/SCID mice received metformin or PBS by oral gavage every day after subcutaneous implantation of RPMI8226 cells. Tumor sizes were monitored every 3 days. **b** Body weight of mice was monitored every 3 days. **c** Immunohistochemical analysis of tumors from the metformin and control groups showing upregulation of AMPK and downregulation of mTOR and Ki-67. Expression levels were scored semi- quantitatively as the percentage of positive cells according to the following system: +,< 25%; ++, 25%–49%; +++, 50%–74%; ++++, 75%–100%. All data are expressed as mean ± SD of values from triplicate experiments. *P < 0.05, **P < 0.01 compared with the control group

We further examined the growth-inhibitory effect of metformin in vivo. NOD/SCID mice were administered metformin (250 mg/kg/d) or physiological saline solution after subcutaneous implantation of RPMI8226 cells. Metformin was found to effectively suppressed growth of RPMI8226 xenografts. Tumor size was significantly lower in the treatment cohort compared with that in the control cohort (Fig. 6a). The weight of the mice was monitored to evaluate possible side-effects caused by metformin. As shown in Fig. 6b, metformin treatment had no significant effect on the body weight of the animals during the course of the treatment. When the treatment was completed after 3 weeks, tumors were resected from mice. Immunohistochemical staining of AMPK, mTOR and Ki67 showed that p-AMPK expression was upregulated compared with that in the control group, whereas expression of both p-mTOR and Ki67 was downregulated (Fig. 6c).

## Discussion

Metformin is a widely used anti-diabetic drug that has recently been shown to exhibit anti-cancer properties both in vitro and in preclinical studies [3–7]. In myeloma, metformin reduces the risk of progression from MGUS to MM and improved outcomes in myeloma patients with diabetes [8, 9]. Previous studies have shown that the cellular and molecular mechanisms responsible for the actions of metformin are differ from cell line to cell line; also, a possible involvement or non-involvement of AMPK pathway has been described [6, 7, 12, 13]. In uterine serous carcinoma (USC), the anti-proliferative action of metformin is mediated by suppression of the IGF-1 receptor pathway [20]. In pancreatic cancer cells, the cytotoxic effect of metformin is mediated by inhibition of both the mTORC1 and ERK pathways [21]. In primary effusion lymphoma (PEL) cells, metformin inhibits both mTOR and STAT3 pathways by decreasing intracellular ROS levels [22]. However, in myeloma, prior to the present study, the cellular and molecular mechanisms have not yet elucidated.

In present study, we showed that metformin repressed both mTORC1 and mTORC2 signaling pathways by activation of AMPK in myeloma cells, and was also induced autophagy and cell-cycle arrest. The mTOR pathway is frequently reported to be involved in tumor survival and progression via two different functional protein complexes, mTORC1 and mTORC2. The first generation mTOR inhibitors (mTORC1 inhibitors), rapamycin, and its analogs are not clinically effective when used as a single agent. It is possible that the efficacy of these agents may be partially restricted by their failure to prevent activation of AKT, an effect that is mediated by a negative feedback loop of the mTORC1/S6 K1/IRS/PI3K axis [23, 24]. It has been shown that dual mTORC1/2 inhibition mediated by agents such as CC-223 and pp432 is associated with much more effective anti-cancer activity than that associated with mTORC1 inhibition alone [25], due to the negatively regulation of AKT phosphorylation at Ser473, which is the direct downstream target of mTORC2 [23]. Nevertheless, these drugs are still in preclinical trials and far from being released.

Interestingly, our study showed metformin represses mTORC1 and mTORC2 simultaneously and is therefore implicated as a potential specific dual mTORC1/2 inhibitor. Furthermore, since hyperglycemia is the most common treatment-related adverse event associated with both first and second generation mTOR inhibitors, metformin has the additional advantage of being licensed as a safe and effective hypoglycemia treatment. The results of the present study indicate that metformin inhibits mTORC1 pathway, with downregulated expressing of the p-mTOR (Se-2448), p-P70S6K and p-4EBP1, which results in the eventual inhibition of mRNA translation and cell proliferation. Repressing of the mTORC2 pathway was also confirmed by Western blot analysis. Expressing of p-mTOR (Ser2481) was downregulated after metformin treatment, and as a direct target of mTORC2, p-AKT (Ser473) was also repressed, leading to enhance inhibition of cell proliferation. Flow cytometric analysis showed metformin induced G0/G1 phage cell cycle arrest in myeloma cells, but no significant apoptosis was observed. These observations are consisted with those of several previous studies in which metformin alone was not found to induce apoptosis in some tumor cell lines [18, 26, 27]. Of note, Jagannathan et al. reported that treatment of myeloma cells with metformin alone did not promote apoptsis, while apoptosis was increased by co-treatment with bortezomib [28].

Autophagy can contribute to cell death, but also served as a survival mechanism for cancer cells. Granato et al. reported that metformin restores autophagy when blocked by bortezomib treatment in PEL cells [22], while another study suggested that metformin suppresses GRP78-dependent autophagy by enhancing the effect of bortezomib in myeloma [28]. In our study, TEM observations showed accumulation of autophagosomes in metformin-treated myeloma cells and expression of the Atg1/ULK1 complex, the downstream target of mTORC1 and the central regulator of autophagy, was found to be simultaneously elevated. When pre-treated with the autophagy inhibitor 3-MA, the inhibitory effects of metformin on myeloma cells were attenuated. This observation confirmed that the activation of autophagy is partially responsible for the inhibition of myeloma cell proliferation.

In this study, both pharmacologic and molecular knock-down of AMPK abrogated metformin-induced myeloma cell growth inhibition. This confirmed that metformin inhibits myeloma cell growth via an AMPK-dependent mechanism. Metformin was previously shown to activate AMPK and phosphorylate TSC2, leading to inactivation of Rheb and mTORC1 [12–14, 29]. In the present study, we demonstrated that metformin represses both the mTORC1 and mTORC2 signaling pathways. However, the involvement of metformin activation of AMPK in the inhibition of the mTORC2-AKT pathway remains to be elucidated. In this regard, some recent studies have shown that TSC2 is necessary and sufficient for mTORC2 association [30, 31], although further investigations are required to thoroughly address these open questions.

In this study, we also confirmed the effectiveness of metformin and the associated changes in the AMPK/mTOR pathway in a NOD/SCID mouse MM xenograft model. The results show that metformin administered at a dose of 250 mg/kg was safe and effective for the treatment of xenografted tumors in mice. A previous report showed that metformin administered at a dose of less than 500 mg/kg in mice yielded plasma levels of metformin similar to those in diabetic patients treated with metformin [32].

## Conclusions

In summary, our findings indicate that metformin inhibits the proliferation of myeloma cells by autophagy induction and G0/G1 phase cell cycle arrest via a mechanism that might involve dual repression the mTORC1 and mTORC2 pathways mediated by AMPK activation. Thus, metformin is implicated as a promising drug for the treatment of myeloma.
